# Structure and Functions of NDR1/HIN1‐Like (NHL) Proteins in Plant Development and Response to Environmental Stresses

**DOI:** 10.1111/pce.15569

**Published:** 2025-04-21

**Authors:** Victoria Amato, Shantel Mahalath, Liyuan Zhang, Paul J. Rushton, Qingxi J. Shen

**Affiliations:** ^1^ School of Life Sciences University of Nevada, Las Vegas Las Vegas Nevada USA

**Keywords:** abiotic stress, Arabidopsis, biotic stress, intrinsic disordered region, LEA2 domain, NHL (NDR1/HIN1), *Oryza sativa* (rice), transmembrane domain

## Abstract

The NON‐RACE‐SPECIFIC DISEASE RESISTANCE 1/harpin‐induced 1‐LIKE (NHL) gene family plays pivotal roles, including pathogen resistance, abiotic stress tolerance, and developmental regulation, underscoring their functional versatility in developmental and physiological processes of plants. NHL proteins often localize to the plasma membrane and contain conserved motifs, including the LEA2 and transmembrane domains, enabling dynamic interactions with signalling molecules and transcription factors. The ability of NHL proteins to dimerize and oligomerize further enhances their regulatory potential in signalling pathways. This review explores the structural and functional diversity of NHL proteins including their localizations, interacting proteins, and responses to abiotic and biotic stresses, ion transportation, seed germination, and responses to phytohormones. Future research integrating phylogenetics, and advanced tools including artificial intelligence will unlock the full potential of this gene family for breeding climate‐resilient crops and agricultural sustainability.

## Summary statement

This review comprehensively explores the NDR1/HIN1‐like (NHL) gene family, highlighting recent advances in structural features, grouping, and mechanistic insights into their diverse functions in biotic and abiotic stress resilience while also proposing their potential as targets for improving crop resilience under climate change challenges.

## 1 Introduction

Climate change is a serious, ongoing global issue that causes a significant impact on numerous aspects of the environment, including plant health. With the increasing rate of climate change, the frequency and intensity of drought events are expected to increase, leading to negative consequences for our crops. Also, the ‘plant disease triangle’ concept introduced by Russell B. Stevens emphasized the three conditions that are required for plant disease development: a susceptible host plant, a virulent pathogen, and favourable environmental conditions (Stevens 1960). Drought stress resulting from climate changes can hence increase the susceptibility of plants to pathogens (for recent reviews, see Jones et al. 2024; Roussin‐Léveillée et al. 2024). The complex relationship between climate change and its effects on drought stress and diseases in plants (Choudhary and Senthil‐Kumar 2024; Rossi et al. 2024; Tissink et al. 2025) therefore emphasizes the critical need for comprehensive research and mitigation strategies to protect crops and ensure food security in this precipitously changing environment.

The *NON‐RACE SPECIFIC DISEASE RESISTANCE‐1* (*NDR1*) gene was originally isolated in *Arabidopsis thaliana* and initially recognized for its role against *Pseudomonas syringae* (Century et al. 1995). NDR1 functions in two major immune strategies: Pathogen‐associated molecular patterns (PAMP)‐triggered immunity (PTI) and effector‐triggered immunity (ETI), acting as a critical intermediary in signalling pathways that initiate and enhance the plant's immune response (for review, see Peng et al. 2018).

Harpin, a heat‐resistant protein that is rich in glycine but lacks cysteine, was first identified in *Erwinia amylovora*, a bacterial pathogen responsible for fire blight in pear, apple, and other members of the Rosaceae family (Wei et al. 1992). Harpin triggers a hypersensitive response (HR) in Arabidopsis, which subsequently leads to the activation of systemic acquired resistance (SAR) driven by salicylic acid (SA). SAR is marked by the upregulation of pathogenesis‐related (PR) genes and other related antimicrobial genes. Some of these antimicrobial genes were initially identified in tobacco and tomato and were referred to as *HIN* (*harpin‐induced*) genes. A stable coiled coil N‐terminal end domain harpin protein, N21_Hpa1_, can cause a HR by upregulating genes involved in SA, jasmonic acid (JA), and ethylene (ET) responses as well as genes associated with ROS production (Ji et al. 2020).

The *NDR1/HIN1‐like* (*NHL*) gene family was first reported in Arabidopsis (Dörmann et al. 2000). In the past decade, these proteins have been shown to play a pivotal role in modulating plant immunity, particularly through the activation of SA‐dependent pathways. In addition, recent studies reveal their roles in plant defence against abiotic stresses, such as drought, and in plant development, for example, seed germination. This review summarizes progress in the study of their grouping, domain and motif structures, and their functions in disease resistance, plant development and response to environmental cues.

## 2 NHL Genes Are Classified Into Six Types Based on Their Motif Combinations

Initially, 29 NDR1/HIN1‐like proteins were discovered in Arabidopsis and were labelled NHL1‐29 (Dörmann et al. 2000). However, subsequent analyses of the Arabidopsis genome revealed that the plant possesses 44 members, including NDR1 (Zheng et al. 2004), and 45 NHL genes (Bao, Song, Pan, et al. 2016). The NHL protein family has been divided into various groups based on sequence homology and phylogenetic analysis (Dörmann et al. 2000; Zheng et al. 2004). A phylogenetic analysis of 59 identified NHL proteins in soybean showed they can be grouped into five groups (Zhang et al. 2022). In these studies, the NHL genes were grouped based on their phylogenetic relationships. However, we found that it is impossible to group NHL genes based on their phylogeny for reasons detailed below.

The study of rice NHLs was first reported in 2013 (Shin 2013). Our latest research identified 76 rice NHL proteins (Table S1). Constructions of phylogenetic trees were attempted using maximum likelihood (ML) and neighbour‐joining (NJ) methods in MEGA 11. The phylogenetic analyses were performed on three sets of sequences: full‐length NHL protein sequences, trimmed protein sequences containing only the transmembrane (TM) domain (see Section 3.1 below), and trimmed protein sequences containing only the late embryogenesis abundant 2 (LEA2) domains (see Section 3.3 below). Furthermore, bootstrap analyses were conducted with iteration counts of 100 and 500 to increase the robustness of phylogenetic relationships. Despite these efforts, the resulting phylogenetic trees exhibited consistently single digit bootstrap values at all primary nodes and most secondary nodes. Similar observations were found for the LEA2 domain trees. These data indicate a lack of confidence in the phylogenetic relationships of the NHL proteins.

**Figure 1:**
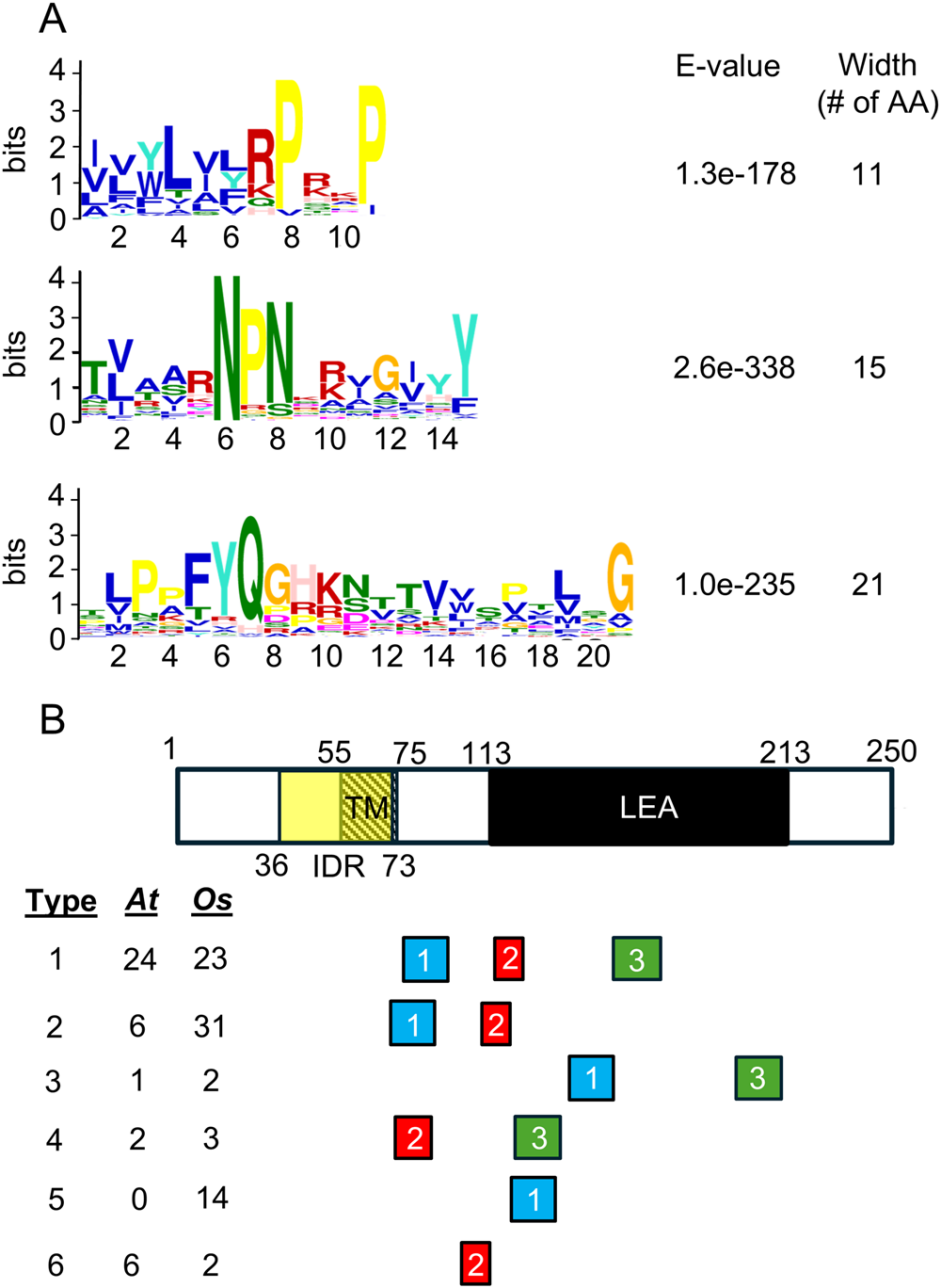
Motifs, domains and types of NHL proteins. (A) Three top motifs (RPX2P, NPN and FYQG) in NHL proteins defined by MEME from combined sequences of *Arabidopsis thaliana* (At) and rice (*Oryza sativa* subsp. *japonica*, Os). AA: amino acid. (B) Domains and types of NHLs. Locations of motif 1: RPX2P (in blue), motif 2: NPN (in red), and motif 3: FYQG (in green) are shown. The position of each motif represents the average position of that motif in both AtNHLs and OsNHLs. The proteins are grouped into six types based on the presence of different motifs. Protein length, domain positions and motif positions are drawn to scale.

Due to the inability to group the gene family into monophyletic groups, the NHL genes in Arabidopsis and rice were categorized based on the presence of their motifs that were defined in MEME (Bailey et al. 2009). These proteins share two highly conserved motifs: motif 2 (**NPN**KRIGIYYD) and motif 3 (P**FYQG**HKN), along with a less conserved sequence known as motif 1 (LILWLIL**RP**XK**P**KFXVQDATV) (Figure 1A). Based on the motif structures, the NHL proteins were classified to six types: Type 1 NHL proteins contain motifs 1, 2 and 3. Type 2 contains motifs 1 and 2. Type 3 contains motifs 1 and 3. Type 4 contains motifs 2 and 3. Type 5 contains only motif 1. Type 6 contains only motif 2 (Figure 1B and Table S1).

## 3 Domains in NHL Proteins

### 3.1 TM Domains

**Figure 2:**
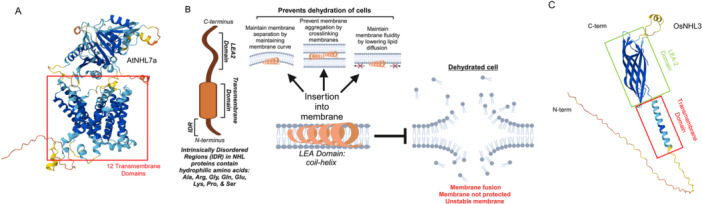
Structural and functional representation of domains in NHL proteins. (A) Predicted structure of AtNHL7a showing 13 predicted transmembrane domains using AlphaFold3. (B) Graphical representation of the mechanisms of the LEA2 domain in NHL proteins to prevent dehydration of cells. IDR contains hydrophilic residues and is located on the intracellular N‐terminal domain of NHL proteins. (C) Predicted 3D structure of OsNHL3 using AlphaFold3. Boxed in green is the LEA2 beta barrel and boxed in red is the alpha‐helix transmembrane domain. [Color figure can be viewed at wileyonlinelibrary.com]

The NHL protein family is characterized by the presence of a predicted TM domain, as revealed by sequence analyses (Figure 1B and Table S1). Most AtNHLs contain only one predicted TM, however, 10 were found in AtNHL7a, 2 in AtNHL16, and 3 in AtNHL41 as revealed by an analysis using a HMM‐based algorithm, TMHMM (Bao, Song, and Zhang 2016; Krogh et al. 2001). However, an artificial intelligence (AI)‐based program, DeepTMHMM (Hallgren et al. 2022), identified 13 TMs in AtNHL7a (Figure 2A and Table S1), 1 in AtNHL16, and 1 in AtNHL41. Alphafold 3, which integrates sequence and structural predictions to model TM topology (Abramson et al. 2024), was performed using default parameters on the AlphaFold Server and confidence scores (pLDDT) to validate TM regions in AtNHL7a. This structural model revealed 12 TMs in AtNHL7a. Proteins with 12 TM domains include transporters for sodium‐driven sugar transporters, various neurotransmitters, nucleosides, osmolytes and basic amino acids (Kilty and Amara 1992). It will be interesting to see if AtNHL7a has a potent ion transport activity. Interestingly, no TM was predicted for AtNHL29.

There is experimental evidence clearly showing the localization of some NHL proteins to the plasma membrane. NDR1 is involved in cell wall‐plasma membrane adhesion, drawing parallels to mammalian integrins (Knepper et al. 2011a). AtNHL3 protein, when tagged with a c‐myc or HA epitag, is localized to the plasma membrane (Varet et al. 2003). The *Capsicum annuum* protein, CaNHL4, was shown to localize to the plasma membrane when transiently expressed (Liu et al. 2020).

### 3.2 Intrinsically Disordered Region (IDRs)

IDRs are segments of a protein that lack a stable and well‐defined three‐dimensional structure under physiological conditions (reviewed in Holehouse and Kragelund 2024). Due to this, IDRs have a high degree of flexibility and dynamic conformations resulting from the inability to form consistent secondary and tertiary structures. This flexibility allows them to swiftly adapt and respond to external conditions. Some studies have highlighted how these IDRs can enable proteins to engage in transient and dynamic interactions within signalling pathways and biological responses to environmental changes. IDRs interact with a variety of molecules including proteins, DNA, RNA, lipids, metal ions and carbohydrates. Their interactions with RNA are particularly important as they can influence posttranscriptional regulation, such as mRNA stability, mRNA localization, or translation efficiency. Many of the IDR interactions with RNA were experimentally determined through a variety of approaches such as: nuclear magnetic resonance (NMR) spectrometry, single‐molecule Forster Resonance Energy Transfer (smFRET), hydrogen‐deuterium exchange mass spectrometry, electrophoretic mobility shift assays (EMSAs), and crosslinking studies. Studies examining IDR interactions with proteins remain limited. However, research into IDR and protein interactions is gaining momentum with recent studies beginning to employ crosslinking mass spectrometry, co‐immunoprecipitation, yeast two‐hybrid screening, and computational prediction to identify IDR and protein interaction (Kibar and Vingron 2023).

In the context of NHL proteins, IDRs are predicted in several members of the family (see Table S1). Some of these disordered regions overlap with TM regions (Figure 1B), which may allow NHL proteins to interact with membranes in a dynamic manner, potentially regulating membrane‐associated processes or interacting membrane‐bound partners.

### 3.3 LEA2 Domains

LEA proteins, also called hydrophilins, are hydrophilic, rich in glycine, and abundant during late embryogenesis. They play protective roles in plant responses to abiotic stress conditions, including drought, salinity, cold and oxidative stress likely through key processes such as (i) preventing denaturation of proteins, (ii) binding metal ions, hence reducing the formation of reactive oxygen species (ROS) and (iii) maintaining membrane integrity (reviewed in Hernández‐Sánchez et al. 2022). Among nine groups of LEAs, that is, LEA1 through 6, dehydrin, seed maturation proteins (SMP), and AtM; LEA2 proteins are the largest group in plants (Jin et al. 2019). They are most abundant in mature seeds but also present in vegetative tissues under both abiotic and biotic stresses. Being intrinsically disordered proteins, LEA2 can change transition between various conformations in reaction to stress conditions through processes that include coil to helix transitions, sustaining membrane fluidity, preventing membrane fusion, and avoiding vesicle aggregation during dehydration by forming molecular shields through membrane cross‐linking (Figure 2B). This transition could sustain membrane fluidity by regulating the phase transition temperature of lipids.

NHL proteins generally contain both a TM domain and LEA2 domain (Figure 1B and Table S1); however, NDR1 contains a TM domain, but no LEA2 domain. It is possible that the structure of NDR1 is protected by LEA2 domains in other NHLs that interact with NDR1 under stress conditions. This structure of NDR1 doesn't seem to be evolutionarily conserved because all OsNHL proteins contain LEA2 domains, as determined by InterPro (Blum et al. 2021) (Table S1). Utilizing AlphaFold3 (Abramson et al. 2024), we predicted the three‐dimensional structure of 76 OsNHL proteins, with all OsNHL proteins containing an alpha‐helical TM domain and a beta barrel structure with the exceptions of OsNHL40, which lacks a beta barrel, and OsNHL71, which misses an alpha‐helix and a beta barrel. Figure 2C illustrates the representation of the NHL protein OsNHL3 in rice, highlighting the alpha‐helical TM region in red and the LEA2 beta barrel in green.

## 4 Functions of NHL Proteins

NHL proteins play diverse roles in plant development and physiological processes, especially plant responses to biotic and abiotic stresses, as summarized in Table 1 and detailed below.

**Table 1:** Functions of NHL proteins in different species.

Species	# of NHL	Gene	Function	Mutation	Reference
*Arabidopsis thaliana*	45	*NDR1*	Activation of CC‐NB‐LRR; cell wall‐plasma membrane adhesions; pathogen resistance at high temperatures	KO, OE	Century et al. (1995), Knepper et al. (2011a), Samaradivakara et al. (2022)
		*AtNHL3*	Scaffold between PDLP5 and CALS1—positive regulator of callose deposition; Interacting with AtOZF1	KO, Transient OE	Singh et al. (2018), Tee et al. (2023)
		*AtNHL6*	ABA hypersensitivity; positive regulator of ABA‐mediated germination; interacting with AtTLP11 for proteolytic degradation	KO, OE	Bao, Song, Pan, et al. (2016), Song et al. (2019)
		*AtNHL12*	Interacting with AtNHL3 to displace interaction between PDLP5 and CALS1—negative regulator of callose deposition	KO, OE	Ayyoub et al. (2024)
		*AtNHL26*	Located in plasmodesmata, regulating plasmodesmata closure and solute movement restriction	OE	Vilaine et al. (2013)
		*AtNHL3 and AtNHL25*	Involved in SA‐dependent (*AtNHL25*) and SA‐independent pathways (*AtNHL3*)	N/A	Varet et al. (2002)
*Lotus japonicus*	N/A	*LjNHL13a*	Interacting with NFR1; assisting in the nodulation process	KO	Yamazaki et al. (2022)
*Solanum tuberosum (Potato)*	N/A	*StPOTHR1*	Enhancing resistance against *Phytophthora infestans*	KD, OE	Chen et al. (2018)
*Triticum aestivum*	N/A	*TaNHL10*	Interacting with ToxA and inducing necrosis in wheat carrying *Tsn1* susceptibility gene	VIGS	Dagvadorj et al.(2022)
*Gossypium hirsutum*	78	*GhNHL69*	Cold stress resistance	KD	Guo et al. (2023)
*Capsicum annuum* L.	15	*CaNHL4*	Enhancing resistance against *Phytophthora capsica*, *Pseudomonas syringe* and Tobacco mosaic virus; facilitating SA and JA pathways; ROS production	Transient OE, VIGS	Liu et al. (2020)
*Brassica napus*	N/A	*BnNHL18A*	Containing SLN‐like sequence for ER retention; moving to cytosol under stress	Transient expression	Lee et al. (2006)

Abbreviations: KD, knockdown; KO, knockout; OE, overexpression; VIGS, virus‐induced gene silencing.

### 4.1 The Role of NHLs in Plant Response to Biotic Stress

#### 4.1.1 NDR1 Mediates Both PTI and ETI

**Figure 3:**
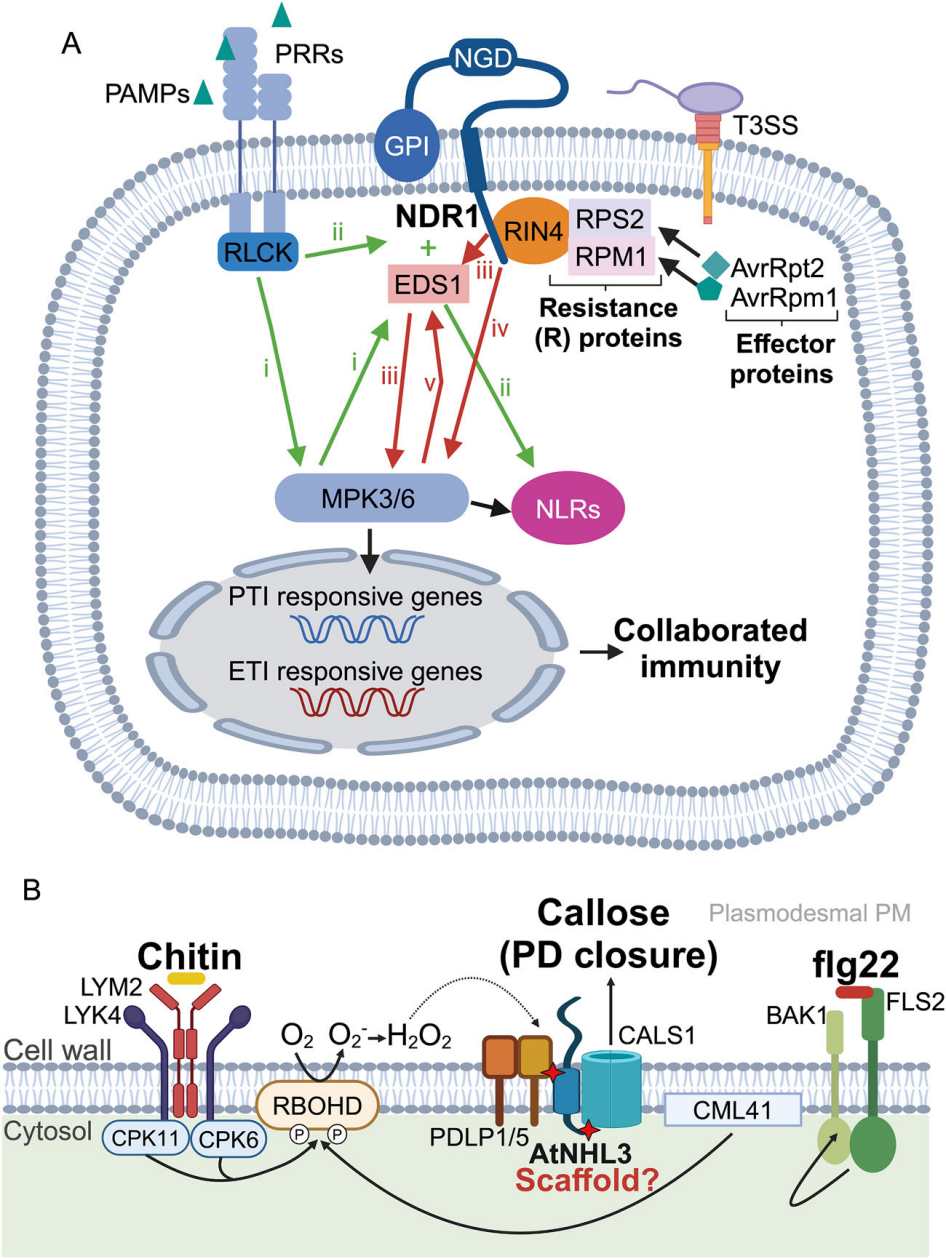
Function of NDR1 and other NHLs in PTI and ETI responses. (A) The role of NDR1 in PTI and ETI. NDR1 protein is localized on the plasma membrane. Recognition of PAMPs by PRRs, triggers signal transduction via receptor‐like cytoplasmic kinases (RLCKs). NDR1 and EDS1 are activated dependent (green lines i) or independent (green lines ii) on the MPK3/6 activities, leading to the upregulation of NLRs proteins. In ETI, NDR1 interacts with RIN4, which is associated with RPS2 and RPM1, to facilitate ETI in response to the effectors AvrRpt2 and AvrRpm1 secreted by the bacteria through a type III secretory system (T3SS). This interaction initiates a signalling pathway involving with (red lines iii) or without (red line iv) EDS1 and MPK3/6, to further increase the expression of NLRs. A positive feedback loop (red line v) between downstream MPK3/6 and EDS1 contributes to the sustained activation of MPK3/6. These activated NLRs trigger ETI, which potentiates and restores PTI through upregulation of PTI components. (B) Illustration demonstrating the crucial role of AtNHL3 in regulating callose deposition in response to chitin and flg22 treatment. LysM‐containing GPI‐anchored protein 2 (LYM2) and LysM domain receptor‐like kinase 4 (LYK4) is a membrane‐bound chitin‐triggered plasmodesmal closure protein and a chitin signalling protein, respectively. CPK11 and CPK6 are calcium‐dependent protein Kinases that phosphorylate respiratory Burst oxidase homologue D (RBOHD). Flagellin‐sensing 2 (FLS2) is perceived by flg22 and causes a downstream signal by interacting with BRI1‐associated kinase 1 (BAK1). Activation of calmodulin‐like 41 (CML41) indirectly leads to phosphorylation of RBOHD. RBOHD converts O_2_ to H_2_O_2_. H_2_O_2_ then acts to close stomata by facilitating the interaction between Plasmodesmata‐located protein 1/5 (PDLP1/5), AtNHL3, and callose synthase 1 (CALS1), with AtNHL3 acting as a scaffold protein. [Color figure can be viewed at wileyonlinelibrary.com]

*NDR1*, the most well‐studied NHL gene, encodes a plasma membrane protein with a glycophosphatidyl‐inositol (GPI) anchor in the C‐terminus (Century et al. 1997; Peter Coppinger et al. 2004). Its functions and signalling pathways are summarized in Figure 3A. The structure of NDR1 revealed similarity to LEA14 (Knepper et al. 2011a), however, the sequence alignment indicated that there is no conserved LEA domain in the NDR1 protein. In addition, NDR1 exhibited structural similarity to mammalian integrins (Knepper et al. 2011a, 2011b). An NGD (Asn‐Gly‐Asp) motif at 178 to 180 amino acids on the C‐terminus is critical for adhesion between the cell wall and plasma membrane. NDR1 formed dimers or oligomers as determined by a co‐immunoprecipitation pull down assay. The NGD motif is not required for the NDR1‐RIN4 association, and the putative function of dimerization of NDR1 in defence signalling is unknown (Knepper et al. 2011a).

Although the immune receptors of PTI and ETI utilize early signalling components and different activation mechanisms, PTI and ETI share certain downstream responses (Yuan et al. 2021). A recent study demonstrated that in Arabidopsis the nucleotide‐binding domain leucine‐rich repeat containing receptor (NLR)‐dependent ETI response relies on the activation of downstream components which is initiated by PRR‐dependent PTI signalling, including mitogen‐activated protein kinases (MPK) (Ngou et al. 2021). These findings suggested the mutual potentiation of PTI and ETI immunity: PTI responses are essential for optimizing ETI responses, and, in turn, ETI responses enhance the accumulation of PTI components (Yuan et al. 2021).

PAMPs are recognized by pathogen recognition receptors (PRRs) on the cell surface, leading to PTI (Lang et al. 2022). Bacterial Type III effector proteins, such as AvrRpt2 and AvrRpm1, were delivered into the plant cytosol via the type III secretion system (T3SS). Resistance driven by NDR1 is dependent on R‐proteins, RPM1 and RPS2. Interaction of NDR1 with RPM1 interacting protein 4 (RIN4) is required for both RIN4‐RPS2 and RIN4‐RPM1 signalling pathways in response to the bacterial pathogen *P. syringae* in Arabidopsis (Day et al. 2006). A physical interaction between RIN4 and RPM1 was shown by yeast two‐hybrid and that of RIN4 and RPS2 by co‐immunoprecipitation (Mackey et al. 2003; Mackey et al. 2002).Enhanced disease susceptibility 1 (EDS1) (dependent on NDR1) or NDR1 acted upstream of sustained MPK3/6 activities, which further led to upregulation of two NLRs in the context of ETI. Additionally, a positive feedback loop involving the downstream signalling of MPK3/6 and EDS1 provided the continued activation of MPK3/6 (Lang et al. 2022).

#### 4.1.2 Other NHLs Also Have Roles in ETI and PTI

AtNHL3 has been shown to mediate Arabidopsis response to bacterial pathogens. It interacts with oxidation‐related zinc finger 1, AtOZF1, a zinc‐finger protein that positively regulates NPR1‐independent SA signalling at the plasma membrane (Singh et al. 2018). AtOZF1 facilitated the expression of NPR1 when SA was exogenously applied (Singh et al. 2018). A T‐DNA insertion mutant, *ozf1*, showed a reduced amount of callose deposition after a *P. syringae* pv. *maculicola* infection (Singh et al. 2018). Deposition of callose, a β‐(1,3)‐D‐glucan polysaccharide, acts as both a physical and chemical barrier, reinforcing the cell wall at the site of pathogen attack and hence slowing pathogen invasion and spread (see Wang et al. 2021, for a recent review). A potential mechanism could be that AtOZF1 interacts with AtNHL3 at the plasma membrane to regulate NPR1 and thus promote the expression of genes involved in callose deposition.

NHLs also mediate plant responses to fungal pathogens. A necrotrophic effector protein, ToxA, from the fungus *Parastagonospora nodorum* interacts with a chloroplast protein, designated ToxA binding protein 1 (ToxABP1) (Manning et al. 2007). The ToxA–ToxABP1 interaction likely induces oxidative stress by disrupting chloroplast function, leading to localized cell death, that is, the HR (Ciuffetti et al. 2010). An interaction between ToxA and a wheat NHL protein, TaNHL10, is necessary to induce necrosis in wheat cultivars carrying the *Tsn1* susceptibility gene. ToxA was shown to interact with the extracellular C‐terminal LEA2 domain of TaNHL10, suggesting that interaction occurs in the apoplasm. Therefore, it is likely that the extracellular domains of NHL proteins are able to interact with effector molecules and promote quick responses to pathogen attacks (Dagvadorj et al. 2022).

Arabidopsis plants overexpressing soybean *GmNHL1* and *GmNHL8* genes showed resistance against *Heterodera glycines* (McNeece et al. 2017). In potatoes (*Solanum tuberosum*), a specific NHL protein, StPOTHR1, contributes to resistance against *Phytophthora infestans*, the pathogen that causes late blight. Overexpression of *StPOTHR1* enhances resistance by limiting pathogen proliferation, with the gene's expression significantly induced at infection sites. StPOTHR1 is also associated with the MAP kinase signalling pathway through interaction with NbMKK5L, suggesting its involvement in the plant's immune response mechanisms (Chen et al. 2018).

#### 4.1.3 Disease Resistance Mediated by NHLs Involves Plasmodesmata

Plasmodesmata‐located protein (PDLP5) is a receptor‐like, type‐I TM protein involved in plasmodesmal function via MAMP‐ and SA‐triggered plasmodesmal signalling to promote callose deposition, which is crucial for the plant's immune response (Lee et al. 2011). Due to the interaction of NHL3 and PDLP5, it was assumed that NHL3 plays similar roles (Cui and Lee 2016; Lee et al. 2011; Tee et al. 2023). Indeed, Arabidopsis *nhl3* knockout lines were unable to close their plasmodesmata when exposed to chitin and flg22 or in the presence of H_2_O_2_ or SA, similar to *pdlp5* and *cals1* knockout mutants (Tee et al. 2023). Therefore, AtNHL3 is a necessary protein for elicitor‐triggered plasmodesmal responses and promotes callose deposition in plasmodesmata by potentially acting as a scaffold protein that mediated the interaction between PDLP5 and CALS1 (Tee et al. 2023) (Figure 3B).

### 4.2 The Roles of NHL Proteins in Abiotic Stress Response

#### 4.2.1 Callose Deposition Is Abiotic Stress Response

**Figure 4:**
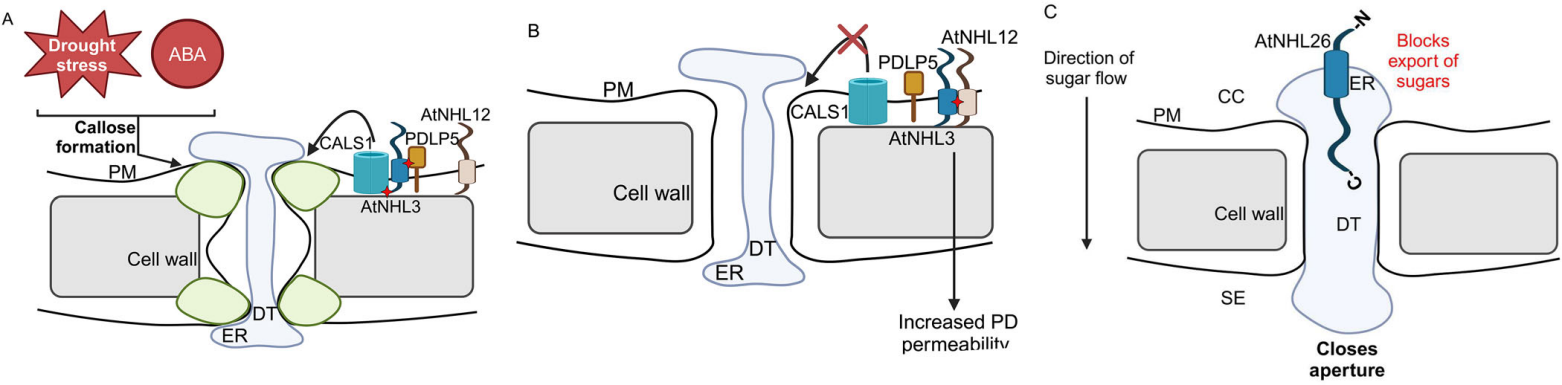
The role of AtNHL3, AtNHL12, and AtNHL26 in callose deposition, plasmodesmatal regulation and abiotic stress. (A) As a scaffold protein, AtNHL3 facilitates the interaction between PDLP5 and CALS1, promoting callose deposition in plasmodesmata. (B) AtNHL12 sequesters AtNHL3 and hence prevents callose formation, increasing permeability between cells. (C) AtNHL26 is localized in the phloem tissue and regulates plasmodesmata aperture size by potentially extending its C‐terminal domain into the desmotubule and blocks export of sugar molecules. Direction of sugar flow indicates sugar flow from source cells to phloem. CC, companion cell; DT, desmotubule; ER, endoplasmic reticulum; PM, plasma membrane; SE, phloem sieve tube elements. [Color figure can be viewed at wileyonlinelibrary.com]

Under those conditions, callose is quickly (within minutes) synthesized and accumulated at the site of stress (Chen and Kim 2009). AtNHL3 facilitates the interaction between PDLP5 and CALS1, promoting callose deposition in plasmodesmata (Figure 4A). AtNHL12 can interact with AtNHL3 at plasmodesmata and thus displaces the interaction of AtNHL3 with PDLP5 and CALS1 and therefore callose deposition is halted, which allows for increased permeability between cells for small molecules (e.g., sugars and hormones) to travel (Ayyoub et al. 2024) (Figure 4B). This indicates that NHL proteins can promote and inhibit callose deposition. Callose depositions under drought may occur by regulating the flow of water in plants (Barzana et al. 2021) and allowing for less transfer of intercellular diffusion of signalling molecules, such as ROS, peptides, and ABA (Takahashi et al. 2020). Moreover, Arabidopsis NHL26 was discovered to be located in the plasmodesmata of phloem cells and desmotubule (DT, a tube that connects the ER of two adjacent plant cells) as well as borders of the companion cell and phloem sieve elements (Vilaine et al. 2013). NHL26, being localized in the plasmodesmata in the phloem tissue, allows for sugar molecules to be blocked via export and therefore, suggests that NHL26 is located in the opening of the plasmodesmata (Vilaine et al. 2013). Furthermore, NHL26 is thought to lead to plasmodesmata closure and hence restriction of solute movement between cells, similar to the role of AtNHL3 (Tee et al. 2023; Vilaine et al. 2013). NHL proteins have long C‐terminal ends that extend to the extracellular region. Therefore, NHL proteins may have the ability to extend their C‐terminal end between cells and allow for the regulation of the aperture of the plasmodesmata (Figure 4C).

#### 4.2.2 Translocation of NHLs in Response to Abiotic Stress Treatments

NHL proteins can modulate abiotic stress response through translocating themselves from a membrane to other cellular components. For instance, GFP‐NHL6 is localized only to the plasma membrane under normal conditions. When treated with ABA, NaCl and mannitol, GFP‐NHL6 was released from the plasma membrane into the cytosol of Arabidopsis mesophyll protoplasts (Bao, Song, Pan, et al. 2016). In canola (*Brassica napus*), BnNHL18A has an N‐terminal cytoplasmic domain that is uncleavable and therefore, suggests that the entire protein is translocated elsewhere in the cell (Lee et al. 2006). This translocation could allow AtNHL6 to interact with cytosolic signalling components for stress responses. In plant drought responses, several key signalling proteins are located in the cytosol, including calcium‐dependent protein kinases (CDPKs), MAPKs, ROS signalling network proteins, ABA signalling proteins such as Sucrose non‐fermenting‐1‐related protein kinase 2s (SnRK2s) and protein phosphatase 2C (PP2C) (for a recent review, see Kuromori et al. 2022). Therefore, NHL proteins might serve as critical hubs in stress signalling, dynamically relocating to adapt to cellular and environmental cues.

Translocation of NHL proteins from ER to cytosol also has been reported. In *B. napus*, the residues 16–46 on the N‐terminal region of *B. napus*, BnNHL18A is localized to the ER in plants (Lee et al. 2006). Upon treatments with stress‐inducing chemicals, including NaCl, hydrogen peroxide, ethephon, and SA, BnNHL18A was shown to translocate from the ER to the cytosol of Arabidopsis protoplasts (Lee et al. 2006). The translocation of BnNHL18A from the ER may be due to stress‐responsive chaperone proteins that may interact with the Sarcolipin‐like sequence (SLN) on BnNHL18A, which acts in a similar way to the animal bZIP protein, ATF6 (Haze et al. 1999; Lee et al. 2006). Collectively, these results suggest that NHLs play roles in regulatory mechanisms of protein translocation and allow for adaptation to cellular stress. This could potentially allow them to travel to, and hence, stabilize ion transporters under stress conditions and maintain ion homoeostasis and proper ion balance.

#### 4.2.3 The Role of NHLs in Ion Transportation Across Tonoplasts as a Response to Abiotic Stress

Plants are able to maintain cellular homoeostasis under various stress conditions, including salinity and drought partly via regulation of transporter proteins, such as plasma membrane H^+^‐ATPases, vacuolar ATPases, and vacuolar H^+^‐translocating inorganic pyrophosphatases. The generated proton motive force then drives the transport of Na^+^ via Na^+^/H^+^ antiporters. Furthermore, calcium transporters, Ca^2+^‐ATPases, found in tomatoes are also shuttled across the membrane, specifically the plasma membrane and tonoplast, to maintain homoeostasis during stress (Ferrol and Bennett 1996). SLN in the ER is dependent on and regulates Ca^2+^‐ATPase of fast‐twitch skeletal muscle in animals (Gramolini et al. 2004). With BnNHL18A containing an SLN‐like sequence, it is suggested that BnNHL18A may regulate Ca^2+^‐ATPases in the cell, specifically in the plasma membrane and tonoplast. Therefore, some NHL proteins may be involved in regulating ion channels in plants to aid in stress responses.

### 4.3 The Role of NHL Proteins in Seed Germination

**Figure 5:**
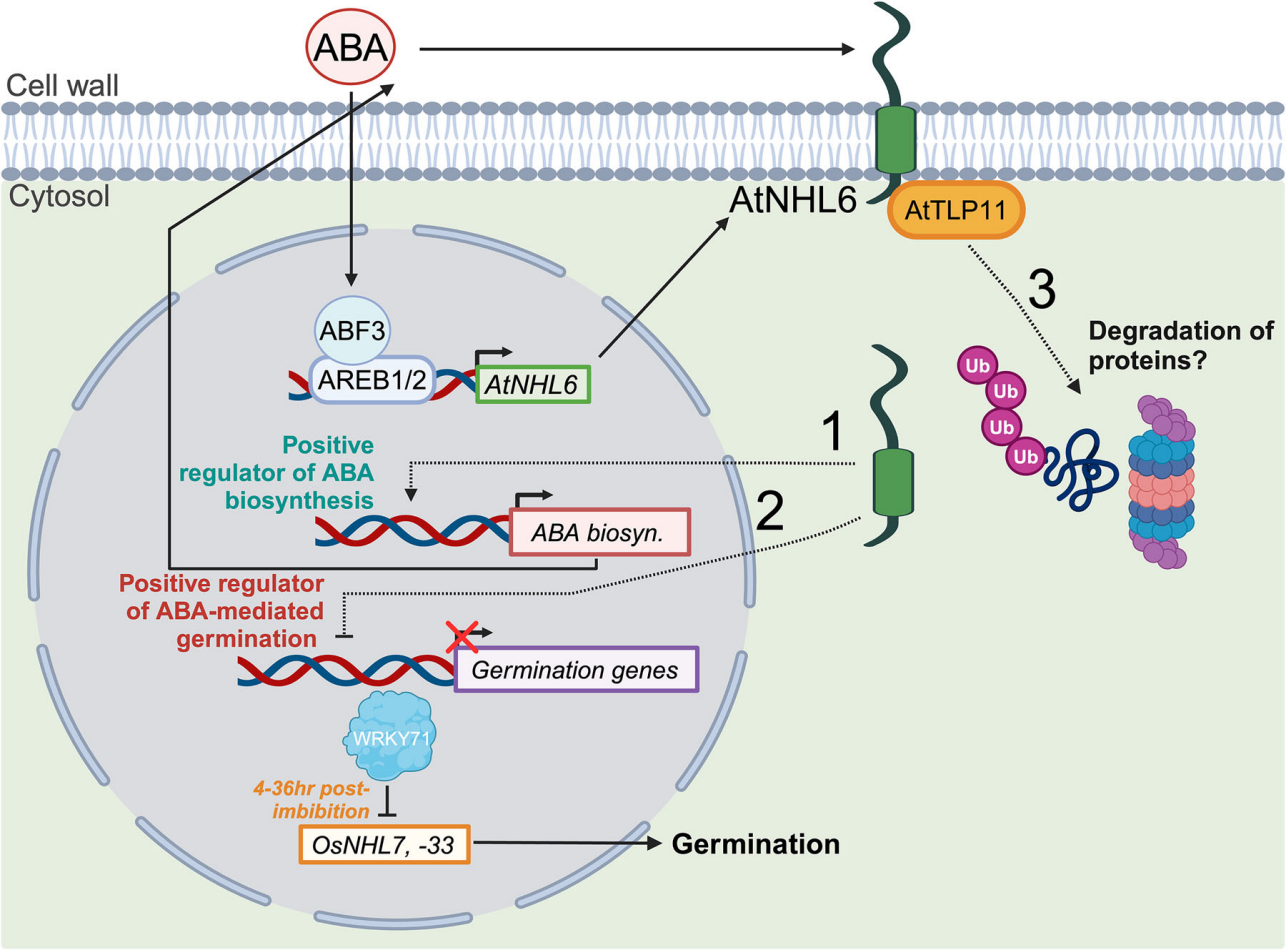
Dynamic role of AtNHL6 in multiple processes and potential regulation of NHL genes in seed germination by WRKY71 in rice. Illustration of the role of AtNHL6 in (1) acting as a positive regulator of ABA biosynthesis genes, (2) acting as a positive regulator on ABA‐mediated germination and (3) interacting with AtTLP11, a tubby‐like F box protein, to aid in the degradation of proteins. OsWRKY71 acts as a negative regulator of seed germination. OsNHL7 and OsNHL33 are negatively regulated by OsWRKY71 at 4–36 h after imbibition. [Color figure can be viewed at wileyonlinelibrary.com]

NHL proteins were also found to be involved in processes in addition to stress responses. AtNHL6 was found to be involved in seed germination (Song et al. 2019). Loss‐of‐function of *NHL6* decreased sensitivity to ABA, a key hormone inhibiting seed germination, in the early developmental stages including seed germination and post‐germination seedling growth (Bao, Song, Pan, et al. 2016). AtNHL6 also acts as a regulator of biosynthesis genes of ABA. The tubby‐like protein family has been shown to mediate ABA signalling and hence implied in controlling seed germination indirectly (Lai et al. 2004). AtNHL6 interacts with AtTLP11, a tubby‐like F‐box protein, on the plasma membrane and thus aids in ubiquitination and proteolytic degradation via 26S proteasome of target proteins to maintain homoeostasis of the proteins (Song et al. 2019). A WRKY transcription factor, OsWRKY71, regulates 36 *OsNHL* genes during seed germination (Bataller et al. 2024). Notably, *OsNHL7* (LOC_Os01g53470) and *OsNHL33* (LOC_Os04g58860) were significantly upregulated in the knockdown mutant, *oswrky71*, from 4 to 36 h after imbibition. OsWRKY71 is a negative regulator of rice seed germination, and these OsWRKY71‐regulated *OsNHL* genes might have a role in rice seed germination. A schematic diagram summarizes the roles of these proteins in controlling seed germination (Figure 5).

## 5 Conclusion and Future Perspectives

### 5.1 Conclusion and Future Perspectives in Basic Research

The NHL gene family represents a diverse and functionally versatile group of proteins that play critical roles in plant responses to both biotic and abiotic stresses and during its development. The large size of the NHL family across different plant species highlights its evolutionary importance and functional specialization, suggesting that this redundancy may provide plants with the flexibility to adapt to varying environmental challenges.

The interaction of NHL proteins with transcription factors, such as the reported relationship between AtNHL3 and AtOZF1, reveals a direct connection between membrane‐localized signals and the transcriptional regulation of stress‐responsive genes. This crosstalk between signalling pathways and gene regulation underscores the role of NHL proteins as key intermediaries in plant responses to environmental stress. Future research should focus on identifying additional NHL‐interacting transcription factors (such as WRKYs), which may uncover novel regulatory mechanisms. Advanced proteomics techniques such as TurboID and split‐TurboID (Cho et al. 2020) could be instrumental in mapping these interactions.

The evolutionary history of the NHL gene family reveals significant diversification, likely driven by the need to adapt to unique ecological niches and stress conditions. However, performing phylogenetic analyses on the NHL family poses challenges likely due to rapid recent evolution of the gene family, as evidenced by low bootstrap values in this study. Leveraging AI and machine learning tools could provide new avenues for robust phylogenetic analyses. AI‐based sequence alignment tools, structural prediction software, and clustering algorithms may help resolve evolutionary relationships that traditional phylogenetics has failed to capture. Such advancements could redefine our understanding of the evolutionary trajectory of this critical gene family.

The NHL family's ability to mediate crosstalk between biotic and abiotic stress responses is a testament to its functional versatility. Increased levels of NDR1 in the overexpression lines recovered the heat‐repressed transcription of ISOCHRISMATE SYNTHASE 1 (ICS1) and CALMODULIN BINDING PROTEIN 60g (CBP60g), therefore, maintained the PTI and SA signalling and led to the improved resistance to Pst DC3000 under raised temperature at 29°C (Samaradivakara et al. 2022). Proteins like AtNHL6 have been shown to influence responses to both pathogen infection and abiotic stresses such as drought and salt stress, potentially by integrating signals from multiple pathways like SA, JA and ABA. Additionally, the role of NHL proteins in regulating ion transport, callose deposition, and plasmodesmatal dynamics further exemplifies their integration into diverse signalling pathways. Future studies should aim to dissect the molecular mechanisms by which NHL proteins mediate these interactions, potentially through multi‐omics approaches combining transcriptomics, proteomics and metabolomics.

### 5.2 Future Applications: Climate‐Resilient Cultivars

The functional versatility of NHL proteins positions them as prime candidates for engineering climate‐resilient cultivars. By overexpressing or modifying specific NHL genes, it may be possible to enhance a plant's tolerance to drought, salinity, and pathogen infection simultaneously. Advanced genomic tools, such as CRISPR‐Cas9/12, could be used to introduce mutations or create allele‐specific edits in NHL genes to fine‐tune their functions. Moreover, identifying natural genetic variations in NHL genes across different crop species and integrating them into breeding programs through marker‐assisted selection or genomic selection could accelerate the development of resilient cultivars.

However, the devil is in the detail in designing strategies to use NHL proteins to improve plants. Increasing stress tolerance by expressing transgenes can come with a fitness cost if this expression happens in the plant in the absence of stress. This makes sense if the plant is devoting energy that could be used for growth into turning on the plants' defences in situations where this may not be necessary. We need to consider the best promoters to use in addition to the NHL transgenes. In many cases, the best promoters will be strongly inducible ones that are induced by the stress that is being targeted (e.g., diseases or drought stress). In such a way, NHL proteins are produced in an enhanced manner only in plant cells that are encountering the stress and this holds the most promise for enhancing stress tolerance (Chen et al. 2018). Similarly, localized RNAi knockdowns using similar inducible promoters could lead to localized reduction in NHL protein levels while having less risk of negative effects (e.g., on germination).

Another general consideration is that of phenotyping the improved plants. Phenotyping can be very subjective and results from one laboratory can be difficult to reproduce in another. Small differences in the growth and measurement conditions can have profound effects on the observed phenotypes of the plants. For this reason, high throughput/precision phenotyping is the best option as it couples noninvasive measurements with high precision of data. Position effects in the greenhouse are also eliminated and experiments can more easily be reproduced. Once improved lines have been well defined, the ultimate goal of improvement under field conditions can be tested.

## Supporting information

Supplemental Table 1. Protein information of the NHL gene family in Arabidopsis thaliana and Oryza sativa subsp. japonica.

## Data Availability

The data that supports the findings of this study are available in the Supporting material of this article.
