# Supplementary material for: Structure and Functions of NDR1/HIN1‐Like (NHL) Proteins in Plant Development and Response to Environmental Stresses
Source: Plant Cell Environ. 2025 Apr 21;48(8):5897–908. doi: 10.1111/pce.15569 (PMC12223711; doi:10.1111/pce.15569)
Supplement: Supplementary file 1 — Supplemental Table 1. Protein information of the NHL gene family in Arabidopsis thaliana and Oryza sativa subsp. japonica. [file PCE-48-5897-s001.docx]

**Supplemental Table 1. The NHL gene family in *Arabidopsis thaliana*and *Oryza sativa*subsp. *japonica*.**

| **Gene Name** | **Locus #** | **AA Size** | **IDR** | **Transmembrane Domain** | **LEA2 Domain** | **Motif** |
| --- | --- | --- | --- | --- | --- | --- |
| *Arabidopsis thaliana* | | | | | | |
| *AtNHL1* | At3g11660 | 209 | N/A | 18-39 | 74-177 | 1, 2, 3 |
| *AtNHL2* | At3g11650 | 240 | N/A | 58-76 | 110-212 | 1, 2, 3 |
| *AtNHL3* | At5g06320 | 231 | N/A | 51-69 | 103-206 | 1, 2, 3 |
| *AtNHL4* | At1g54540 | 239 | N/A | 57-78 | 112-208 | 1, 2, 3 |
| *AtNHL5* | At1g61760 | 224 | 1-22 | 44-64 | 98-195 | 1, 2, 3 |
| *AtNHL6* | At1g65690 | 252 | 1-46 | 70-90 | 123-222 | 1, 2, 3 |
| *AtNHL7a* | At1g08140 | 818 | N/A | 88-105; 123-143; 156-176; 192-209; 222-244; 251-271; 288-308; 310-319; 340-355; 380-400; 409-430; 439-458 | N/A | N/A |
| *AtNHL7b* | At1g23950 | 373 | N/A | N/A | N/A | N/A |
| *AtNHL8* | At1g32340 | 688 | 1-91; 29-40; 42-62; 66-77 | N/A | N/A | N/A |
| *AtNHL9* | At2g35460 | 238 | N/A | 57-76 | 110-212 | 1, 2, 3 |
| *AtNHL10* | At2g35980 | 227 | N/A | 44-63 | 96-199 | 1, 2, 3 |
| *AtNHL11* | At2g35970 | 211 | N/A | 24-44 | 76-180 | 1, 2, 3 |
| *AtNHL12* | At2g35960 | 210 | N/A | 23-43 | 75-179 | 1, 2, 3 |
| *AtNHL13* | At2g27080 | 260 | 1-35 | 79-99 | 132-235 | 1, 2, 3 |
| *AtNHL14* | At2g27260 | 243 | 1-36; 20-33 | 62-84 | 116-196 | 1, 2, 3 |
| *AtNHL15* | At2g01080 | 231 | N/A | 44-65 | 110-208 | 2, 3 |
| *AtNHL16* | At3g20610 | 222 | N/A | 10-34 | N/A | 1, 3 |
| *AtNHL17* | At3g44220 | 206 | N/A | 20-42 | 74-177 | 1, 2, 3 |
| *AtNHL18* | At3g52470 | 208 | N/A | 21-41 | 73-177 | 1, 2, 3 |
| *AtNHL19* | At4g01410 | 227 | 1-36 | 45-65 | 99-189 | 1, 2, 3 |
| *AtNHL20* | At4g26490 | 268 | 1-24 | 89-113 | 147-238 | 1, 2 |
| *AtNHL21* | At4g05220 | 226 | N/A | 46-66 | 100-202 | 1, 2, 3 |
| *AtNHL22* | At4g09590 | 211 | N/A | 24-44 | 76-180 | 1, 2, 3 |
| *AtNHL23* | At5g06330 | 207 | N/A | 19-41 | 76-179 | 1, 2, 3 |
| *AtNHL24* | At5g22850 | 493 | N/A | N/A | N/A | N/A |
| *AtNHL25* | At5g36970 | 248 | 1-11; 1-54 | 66-86 | 119-220 | 1, 2, 3 |
| *AtNHL26* | At5g53730 | 213 | N/A | 27-49 | 86-186 | 1, 2, 3 |
| *AtNHL28* | At5g11890 | 287 | 1-45 | 87-107 | N/A | 1, 2 |
| *AtNHL29* | At1g17550 | 511 | N/A | N/A | N/A | N/A |
| *AtNHL30* | At1g17620 | 264 | N/A | 66-86 | 119-228 | 1, 2, 3 |
| *AtNHL31* | At1g64440 | 348 | N/A | N/A | N/A | N/A |
| *AtNHL32* | At1g64450 | 342 | 149-249 | 20-40 | N/A | 1, 2 |
| *AtNHL33* | At1g01460 | 427 | 247-287; 334-355 | N/A | N/A | N/A |
| *AtNHL34* | At1g13050 | 317 | 18 - 130; 18-31; 56-65; 111-122 | 141-161 | N/A | 2 |
| *AtNHL35* | At1g70040 | 193 | N/A | 10-30 | N/A | N/A |
| *AtNHL36* | At2g46300 | 252 | 1-48 | 65-85 | 121-217 | 1, 2, 3 |
| *AtNHL37* | At3g52460 | 300 | 1-72; 42-56 | 109-130 | N/A | 1, 2 |
| *AtNHL38* | At3g20590 | 255 | N/A | 18-34 | N/A | 1, 2, 3 |
| *AtNHL39* | At3g54200 | 235 | 1-41; 28-41 | 53-74 | 115-212 | 2 |
| *AtNHL40* | At3g26350 | 356 | 1-167; 17-36; 45-54; 73-82 | 180-200 | 234-335 | 2 |
| *AtNHL41* | At3g24600 | 306 | N/A | 117-138 | 179-282 | 2, 3 |
| *AtNHL42* | At4g01110 | 261 | 1-40 | 69-88 | 129-225 | 1, 2 |
| *AtNHL43* | At5g56050 | 283 | 1-25 | 105-125 | N/A | 1, 2 |
| *AtNHL44* | At5g45300 | 689 | 1-100 | N/A | N/A | N/A |
| *AtNDR1* | At3g20600 | 219 | N/A | 19-34 | N/A | 1, 2, 3 |
| *Oryza sativa* subsp. Japonica * | | | | | | |
| *OsNHL1* | Os01g0195400 | 288 | 1-42 | 63-83 | 120-218 | 1, 2, 3 |
| *OsNHL2* | Os01g0228500 | 330 | 1-105; 1-13; 47-58 | 145-165 | 206-309 | 1, 2, 3 |
| *OsNHL3* | Os01g0234700 | 331 | 1-44; 10-22; 23-42 | 75-95 | 128-243 | 1, 2 |
| *OsNHL4* | Os01g0574800 | 291 | N/A | 81-104 | 137-234 | 1, 2, 3 |
| *OsNHL5* | Os01g0711900 | 204 | N/A | 17-37 | N/A | 2 |
| *OsNHL6* | Os01g0712500 | 168 | N/A | 63-88 | N/A | 1 |
| *OsNHL7* | Os01g0736500 | 251 | 14-59; 43-53 | 78-93 | 134-233 | 1, 2 |
| *OsNHL8* | Os01g0812100 | 312 | 1-90; 23-33; 60-73; 79-90 | 149-171 | 214-318 | 1, 2, 3 |
| *OsNHL9* | Os01g0864300 | 241 | N/A | 54-74 | 114-213 | 1, 2, 3 |
| *OsNHL10* | Os01g0864500 | 238 | N/A | 50-70 | 107-195 | 1, 2 |
| *OsNHL11* | Os01g0909000 | 455 | 1-257; 154-169; 231-249 | 273-293 | 334-431 | 1, 2 |
| *OsNHL12* | Os02g0100600 | 214 | N/A | 33-55 | 84-178 | 1, 2 |
| *OsNHL13* | Os02g0261000 | 290 | 1-82; 1-11; 16-34 | 91-112 | N/A | 1, 2 |
| *OsNHL14* | Os02g0265800 | 201 | N/A | 18-38 | 80-179 | 1, 2 |
| *OsNHL15* | Os02g0507900 | 250 | 12-43 | 55-77 | 121-227 | 1, 2, 3 |
| *OsNHL16* | Os02g0538700 | 224 | N/A | 21-41 | 73-175 | 1, 2, 3 |
| *OsNHL17* | Os02g0666600 | 197 | N/A | 19-39 | 73-175 | 1, 2 |
| *OsNHL18* | Os02g0667600 | 337 | 1-26 | 143-163 | 204-313 | 2, 3 |
| *OsNHL19* | Os03g0191800 | 271 | 1-43; 7-16; 17-26 | 94-114 | N/A | 1, 2 |
| *OsNHL20* | Os03g0216500 | 350 | 68-136 | 156-176 | N/A | 1, 2 |
| *OsNHL21* | Os03g0262700 | 238 | 1-21 | 41-62 | 117-215 | 1, 2, 3 |
| *OsNHL22* | Os03g0696000 | 232 | N/A | 57-74 | 108-202 | 1 |
| *OsNHL23* | Os03g0836300 | 212 | N/A | 21-41 | 78-164 | 1, 3 |
| *OsNHL24* | Os03g0836400 | 209 | N/A | 19-42 | 75-186 | 1 |
| *OsNHL25* | Os04g0114300 | 198 | 145-198 | 9-29 | N/A | 1 |
| *OsNHL26* | Os04g0416700 | 220 | N/A | 19-40 | 72-173 | 1, 2, 3 |
| *OsNHL27* | Os04g0430600 | 490 | 1-108; 13-24; 40-58; 65-82; 83-105 | 147-168 | 209-326 | 1, 2, 3 |
| *OsNHL28* | Os04g0628300 | 222 | N/A | 35-58 | 87-198 | 1, 2 |
| *OsNHL29* | Os04g0677300 | 255 | 1-63 | 74-94 | 129-220 | 1, 2, 3 |
| *OsNHL30* | Os04g0685300 | 205 | N/A | 29-48 | 81-170 | 1, 2, 3 |
| *OsNHL31* | Os04g0685400 | 220 | N/A | 24-45 | 80-184 | 1, 2, 3 |
| *OsNHL32* | Os04g0689500 | 315 | 17-131; 24-35; 78-87; 93-108 | 159-180 | 221-328 | 2 |
| *OsNHL33* | Os05g0199100 | 293 | 1-22 | 67-88 | 125-227 | 1, 2, 3 |
| *OsNHL34* | Os05g0238400 | 233 | N/A | 30-50 | N/A | 1, 2 |
| *OsNHL35* | Os05g0367900 | 260 | 1-15; 1-32 | 56-77 | 122-219 | 1, 2 |
| *OsNHL36* | Os05g0482501 | 211 | N/A | 23-47 | 88-179 | 1, 2 |
| *OsNHL37* | Os05g0526700 | 253 | 1-37 | 61-76 | 120-219 | 1, 2 |
| *OsNHL38* | Os05g0584300 | 247 | N/A | 58-78 | 122-222 | 1 |
| *OsNHL39* | Os06g0116900 | 439 | N/A | 15-39 | N/A | 1, 2 |
| *OsNHL40* | Os06g0118301 | 237 | 101-129; 109-120 | 15-39 | N/A | 1, 2 |
| *OsNHL41* | Os06g0118800 | 241 | N/A | 27-48 | N/A | 1 |
| *OsNHL42* | Os06g0118900 | 221 | N/A | 28-50 | N/A | 1, 2 |
| *OsNHL43* | Os06g0119200 | 199 | N/A | 15-35 | 64-176 | 1, 2 |
| *OsNHL44* | Os06g0119800 | 218 | N/A | 24-48 | N/A | 1 |
| *OsNHL45* | Os06g0119900 | 218 | N/A | 34-55 | 84-189 | 1, 3 |
| *OsNHL46* | Os06g0121000 | 211 | N/A | 20-41 | N/A | 1 |
| *OsNHL47* | Os06g0121650 | 226 | N/A | 23-43 | N/A | 1, 2 |
| *OsNHL48* | Os06g0121400 | 199 | N/A | 17-37 | N/A | 1, 2 |
| *OsNHL49* | Os06g0121700 | 181 | N/A | 2-20 | 51-156 | 1, 2 |
| *OsNHL50* | Os06g0163300 | 322 | 1-101 | 134-154 | 196-300 | 1, 2 |
| *OsNHL51* | Os06g0521300 | 238 | 22-42 | 55-72 | 112-207 | 1 |
| *OsNHL52* | Os06g0710500 | 250 | 26-52; 39-52 | 61-89 | N/A | 1, 2 |
| *OsNHL53* | Os07g0206800 | 257 | 1-30 | 85-101 | 135-232 | 1 |
| *OsNHL54* | Os07g0250501 | 205 | N/A | 16-38 | 70-181 | 1 |
| *OsNHL55* | Os07g0250900 | 227 | N/A | 34-58 | 93-203 | 1 |
| *OsNHL56* | Os07g0524200 | 213 | N/A | 16-36 | 70-169 | 1, 2 |
| *OsNHL57* | Os07g0524400 | 190 | N/A | 13-31 | 69-157 | 1, 2 |
| *OsNHL58* | Os07g0531500 | 200 | N/A | 18-41 | 74-177 | 1, 2, 3 |
| *OsNHL59* | Os08g0102700 | 227 | N/A | 23-43 | 88-191 | 1, 2, 3 |
| *OsNHL60* | Os08g0163600 | 306 | 1-45; 66-114 | 124-144 | N/A | 1, 2 |
| *OsNHL61* | Os08g0324200 | 242 | N/A | 62-78 | 115-216 | 1, 2, 3 |
| *OsNHL62* | Os08g0494000 | 232 | N/A | 52-72 | 112-200 | 1, 2 |
| *OsNHL63* | Os08g0558300 | 192 | N/A | 20-35 | 68-164 | 1, 2 |
| *OsNHL64* | Os09g0267400 | 339 | N/A | 30-47 | 82-184 | 1, 2, 3 |
| *OsNHL65* | Os09g0532200 | 218 | N/A | 22-45 | N/A | 1, 2 |
| *OsNHL66* | Os10g0547200 | 242 | N/A | 22-44 | 108-217 | 1, 2, 3 |
| *OsNHL67* | Os11g0120100 | 235 | N/A | 53-71 | 112-208 | 1 |
| *OsNHL68* | Os11g0130400 | 276 | 1-42 | 89-109 | 143-247 | 2, 3 |
| *OsNHL69* | Os11g0157200 | 210 | N/A | 20-42 | 77-180 | 1, 2, 3 |
| *OsNHL70* | Os11g0157300 | 342 | 39-85; 40-52; 112-126; 127-144 | 157-178 | 212-314 | 1, 2, 3 |
| *OsNHL71* | Os11g0445475 | 133 | N/A | 15-24 | 60-121 | 1 |
| *OsNHL72* | Os12g0119800 | 233 | 1-35 | 51-69 | 110-206 | 1 |
| *OsNHL73* | Os12g0127200 | 275 | 1-45 | 93-110 | 144-248 | 2, 3 |
| *OsNHL74* | Os12g0158900 | 224 | N/A | 16-33 | 76-194 | 1, 2, 3 |
| *OsNHL75* | Os12g0159000 | 192 | N/A | 15-33 | 65-160 | 1, 2 |
| *OsNHL76* | Os12g0159600 | 212 | N/A | 20-42 | 79-182 | 1, 2, 3 |

*: Identified using the Hidden Markov Model (HMM) approach with a cutoff value of 0.001.
